# Carbohydrate restriction ameliorates nephropathy by reducing oxidative stress and upregulating HIF-1α levels in type-1 diabetic rats

**DOI:** 10.1186/s40200-017-0331-5

**Published:** 2017-12-19

**Authors:** Pawan Krishan, Gaaminepreet Singh, Onkar Bedi

**Affiliations:** 1 Department of Pharmaceutical Sciences and Drug Research, Punjabi University, Patiala, Punjab India; 2 JRF, DST-SERB, New Delhi, India

**Keywords:** Diabetic nephropathy, Carbohydrate restriction, Hypoxia, HIF-1 alpha, Oxidative stress, Oxygen consumption (QO_2_), Streptozotocin (STZ) induced Type-1 diabetes

## Abstract

**Background:**

Carbohydrate restricted diet regimen is widely accepted as therapeutic approach for the treatment of kidney disease associated with type-2 diabetes, obesity and hypertensive disorders. The present study tested the influence of carbohydrate-energy restricted diet (CR) on type-1 diabetes induced renal dysfunction, hypoxia and structural alterations against diabetic rat group fed control diet (*ad libitium*).

**Methods:**

Male wistar rats weighing between 180 and 190 g were subjected to 30% carbohydrate energy restricted diet (CR) and diabetes was induced by administration of streptozotocin (45 mg/kg., *i.p*). Assessment of renal function was done after 4 weeks by determining the serum levels of creatinine, BUN, proteinuria. Oxidative stress was determined by estimating the reduced glutathione, malonaldehyde levels, catalase activity and extent of renal hypoxia by estimating the HIF-1α levels in kidney tissue homogenates. Histological studies were conducted on kidney sections using hematoxylin and eosin, periodic acid-schiff staining.

**Results:**

Diabetic rats exhibited marked hyperglycemia and renal dysfunction developed in diabetic rats fed control diet (*ad libitium*) as shown by significantly elevated levels of serum creatinine, BUN and massive proteinuria after 4 weeks period. CR diet treatment in diabetic rats significantly lowered hyperglycemia, reversed the above renal functional abnormalities, reduced oxidative stress and enhanced HIF-1α levels. Furthermore histological examination of kidney sections from CR diet treated diabetic rat group showed absence of glomerular hypertrophy, mesangial expansion and tubular vacoulations.

**Conclusion:**

Our results demonstrated that CR diet treatment in diabetic rats attenuated renal damage by reducing oxidative stress and preventing the development of hypoxia by up-regulating HIF-1α levels.

## Background

Diabetic nephropathy is a common complication in diabetes mellitus and often results in the need for dialysis or kidney transplantation. The excessive ROS production in diabetes is considered as a direct consequence of hyperglycemia [1]. The pathogenesis of diabetic nephropathy also involves strong association between both increased oxidative stress and kidney tissue hypoxia. The enhanced oxidative stress has been suggested to impair tubular electrolyte efficiency and produce mitochondrial derangements in kidney resulting in increased oxygen consumption (QO_2_), and reduced oxygen tension (PO_2_) [2, 3]. The reduced kidney PO_2_ is manifested as hypoxia which can induce immune cell infiltration, proteinuria and tubulointerstitial fibrosis, all considered as pathological hallmarks of chronic kidney diseases [4, 5]. Importantly, the renal hypoxia is now recognised as an independent risk factor for development of nephropathy [6].

HIFs modulate key metabolic pathways to optimize glucose and O_2_ utilization for generation of sufficient amounts of ATP. It is comprised of two different subunits: an α-subunit that is rapidly deteriorated by prolyl hydroxylases in the presence of oxygen and a constitutively expressed β-subunit. During oxygen deprivation in hypoxic sate HIF-1α accumulation occurs which combines with β subunit to form transcriptionally active heterodimer that subsequently remodels the renal hemodynamics by producing blood cells and by inducing formation of new vessels via targeting erythropoietin and vascular endothelium growth factor (VEGF) [7, 8]. In addition a study has demonstrated that pharmacological activation of HIFs by cobalt treatment prevented the diabetes-induced alterations in oxygen metabolism, mitochondrial leak respiration, kidney function and reduced proteinuria and tubulointerstitial damage [9].

The current therapeutic approaches for treatment of diabetic nephropathy includes use of angiotensin-converting enzyme inhibitors, angiotensin-receptor blockers and antihypertensive drugs. However these agents may not be completely effective in halting the progression of diabetic nephropathy. Carbohydrate energy restriction has been reported to improve physical endurance and reduce brain oxidative damage against exhaustive exercise test in rats [10]. Another study has demonstrated that beneficial effects of the low sucrose diet intake on ischemia reperfusion induced renal injury in mice. One week of low carbohydrate dietary preconditioning in mice reduced postoperative weight loss and improved renal dysfunction in mice subjected to bilateral renal ischemic injury [11]. Kume et al., 2010 [12] reported that long term general calorie restriction promotes cellular adaptive mechanisms in response to aging associated renal hypoxia in mice. Furthermore dietary calorie restriction exposure (9 weeks) in diabetic rats was found to exert protective effects by reducing the oxidative damage and inflammatory response in rat kidney [13]. Importantly carbohydrate restriction in humans is now recognised as an efficacious and safe approach for management of diabetes [14, 15]. However, so far no study has explored the impact of carbohydrate restricted diet intake on renal hypoxia, the major pathogenic event leading to end stage renal disease in type-1 diabetes. Furthermore the study also aims to gain insight into the possible influence of carbohydrate restricted diet intake on the functional and structural impairments associated with diabetic rats.

## Methods

The experimental procedures on rats were approved by institutional animal ethics committee of Department of Pharmaceutical Sciences and Drug Research, Punjabi University, Patiala (Punjab), India. Male wistar rats 3 months old weighing 180-190 g were divided into five groups normal control (NC), diabetic control (DC), vehicle control (VC), CR control (Carbohydrate restricted only), CR + STZ (Carbohydrate restricted streptozotocin treated rats). Each rat from all groups was housed individually in cages. NC, DC, VC, groups were maintained on AIN-93 M carbohydrate during entire study period. The normal carbohydrate included: Cornstarch 465.6 g, casein 140 g, dextrinized cornstarch 155 g, sucrose 100 g, soyabean oil 40 ml, cellulose 50 g, l-cystine 1.8 g, choline bitartrate 2.5 g, tert-butylhydroquinone 0.008 g, AIN-93 M mineral mix 35 g, AIN-93 M vitamin mix 10 g, vitamin E 150 mg, vitamin k-0.75 mg for 1 kg carbohydrate (Table 1) [16]. Vitamin premix was stored in air tight zip lock plastic bag and kept at −20˙C until further use. Choline bitartrate was added separately to final carbohydrate due to its hygroscopic nature and vitamin E was used in carbohydrate preparation by mixing in soyabean oil. CR control, CR + STZ groups were exposed to carbohydrate restricted diet 2 weeks prior to start of the experiment and retained on this diet for the remaining period of 4 weeks after streptozotocin injection (45 mg/kg., *i.p*).

**Table 1:** Composition of the two different diets based on AIN-93-M for 1 kg diet [10, 16]

S.No	Ingredients	Control Diet	Carbohydrate Restricted Diet
1.	Cornstarch	465.6 g	408.4 g
2.	Dextrinized cornstarch	155 g	133 g
3.	Casein	140 g	200 g
4.	Sucrose	100 g	72.2 g
5.	Soyabean Oil	40 g	57.1 g
6.	Cellulose	50 g	58.6 g
7.	Mineral Mixture	35 g	50 g
8.	Vitamin Mixture	10 g	14.3 g
9.	L-Cystine	1.8 g	2.6 g
10.	Choline Bitartrate	2.5 g	3.6 g
11.	*Tert*-butylhydroquinone	0.008 g	0.005 g
12.	DL-α-Tocopherol	150 mg	220 mg
13.	Vitamin K1	0.75 mg	1.07 mg

**Fig. 1:**
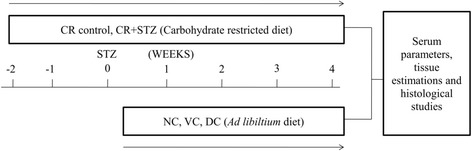
Experimental study design. Carbohydrate energy restriction was initiated 2 weeks prior to the start of study in carbohydrate restricted diet control group (CR control) and streptozotocin treated carbohydrate restricted rats (CR + STZ) group. Diabetes was induced at 0 week time in CR + STZ and DC group. Body weight was taken for each rat every 2 weeks period. After 4 weeks serum, renal tissue biochemical and histological examination was done in each group rats. NC (normal control), VC (Vehicle control), DC (Diabetic control)

Carbohydrate energy restricted diet was composed of cornstarch 408.4 g, casein 200 g, dextrinized cornstarch 133 g, sucrose 72.2 g, soyabean oil 57.1 ml, cellulose 58.6 g, l-cystine 2.6 g, choline bitartrate 3.6 g, tert-butylhydroquinone 0.0055 g, AIN mineral mix 50 g, AIN-93 M vitamin mix 14.30 g, vitamin E 220 mg, vitamin K-1.07 mg for 1 kg carbohydrate (Table 1). Carbohydrate restricted diet was formulated in such a manner when offering a 30% reduced quantity of diet consumed by NC and VC groups this diet offered equivalent quantity of all nutrients (proteins, fats, minerals, vitamins). Thus carbohydrate energy restricted group ingested 40% less carbohydrates compared to NC group [10]. The feed consumed by NC, VC, DC group was monitored daily and average was calculated at 0, 2, 4 weeks of study protocol. Weight changes were recorded weekly from which final mean weights of all groups were obtained. Diabetes was induced by single dose of 45 mg/kg., *i.p* streptozotocin [17] (Sigma Chemicals Co, St Louis, MO, USA). The status of diabetes in rats was confirmed by a tail-blood glucose level higher than 16.7 mmol/l 48 h after STZ injection [18] and further blood glucose levels (tail vein, glucometer) were determined at 0, 2, 4 weeks in all study groups. Blood was collected from retro-orbital plexus of rats under light isoflurane anaesthesia and assessment of renal function was done after 4 weeks by biochemical estimations of blood urea nitrogen (BUN, GLDH-Urease method), serum creatinine (Jaffe’s method), serum and urine albumin levels (BCG Dye method), proteinuria (24 h urine, Biuret method) was done using diagnostic kits from Tranasia Bio-medical Ltd. (Erba Mannheim) on chem-5 plus V2 semi autoanalyzer (Erba Mannheim). Rats were anesthetized by an intraperitoneal injection of sodium pentobarbital [17], and both kidneys were isolated cleaned and weighed to calculate kidney/body weight ratio. Then rats were sacrificed by cervical dislocation and left kidney was taken for preparation of tissue homogenate (10% in cold phosphate buffer saline, pH 7.4). Further estimation of total protein content was done using bovine serum albumin as standard [19]. A detailed schematic representation of the experimental design is depicted in Fig. 1.

### Determination of antioxidant enzyme, lipid peroxidation and HIF-1α levels

The level of lipid peroxidation in kidney homogenate was estimated by measuring the formation of thiobarbituric acid reactive substances (TBARS) [20]. A standard plot was prepared using 1,1,3,3-tetramethoxypropane and values were expressed as nmol of malonaldehyde per milligram protein (nmol MDA/mg protein). Reduced glutathione levels were measured according to method of Boyne and Ellman [21]. A standard curve was plotted using reduced glutathione and results were expressed as micromoles of reduced glutathione per milligram tissue protein (μmol/mg protein). Catalase enzyme activity was determined from analytical method as described by Gwinner et al. [22]. Renal hypoxia was assessed by checking the levels of hypoxia inducible factor-1α in renal tissue by using rat HIF-1α ELISA kit (Code: ER0191, Wuhan Fine Biological Technology Co., Ltd. Wuhan, China) on i-Mark Microplate reader, BIO-RAD.

### Histopathological examination

The right kidney was fixed in 10% formaldehyde solution. After fixation pieces of tissues were embedded into paraffin, cut into 5 μm slices and stained with hematoxylin and eosin stain to examine the extent of renal damage or protection and observed at 100X magnification (Leica DM 4000 microscope). Periodic acid Schiff staining was performed on 4 μm thick kidney sections to analyse the glomerular injury and observed at 400X magnification.

### Statistical analyses

Statistical analyses were performed using Graph pad prism software-5 by applying one way ANOVA followed by bonferonni post hoc test for multiple comparison between groups. Data are shown as mean ± SD of *n* = 30, 6 per group. A *p*-value less than 0.05 (*P* < 0.05) was considered as statistically significant.

## Results

### Effects of carbohydrate restriction on body weight changes in various study groups

**Fig. 2:**
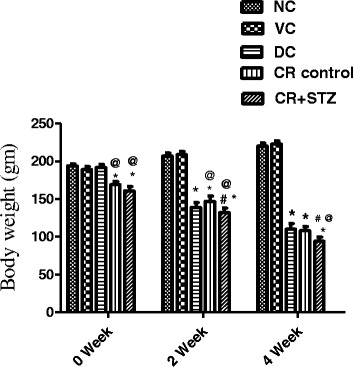
Effects of carbohydrate restricted diet on body weight changes in streptozotocin induced diabetic rats at 0, 2, 4 weeks of treatment. NC: normal control, VC: vehicle control, DC: diabetic control with streptozotocin administration only, CR control: carbohydrate restricted diet treatment in normal rats, CR + STZ: carbohydrate restricted diet treatment in streptozotocin administered rats. Data are shown as mean ± SD. A *P*-value less than 0.05 (*P* < 0.05) was considered statistically significant. * Vs Normal control and vehicle control, ^@^ Vs Diabetic control, ^#^ Vs CR control

Body weight was found to be significantly reduced at the beginning of zero week in carbohydrate restricted control and streptozotocin treated carbohydrate restricted groups in comparison to normal control, vehicle control and diabetic control groups (Fig. 2). During second week diabetic control group showed a significant decline of body weight 32.86% and 33.50% (*p* < 0.001) compared with normal and vehicle control group also. The decline in body weight at second week among diabetic control, carbohydrate restricted control and carbohydrate restricted streptozotocin treated rat groups was found to be persistent towards fourth week of study also (Fig. 2). Importantly the body weight change in calorie restricted streptozotocin treated group was noticed to be significantly lower compared to carbohydrate restricted control group (*P* < 0.001) at both second and fourth week of study period.

### Effects of carbohydrate restriction on kidney weight/body weight ratio in diabetic rats

**Fig. 3:**
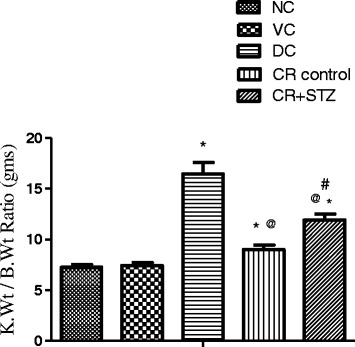
Effects of carbohydrate restricted diet on kidney weight/body weight ratio*1000 in streptozotocin induced diabetic rats after 4 weeks. NC: normal control, VC: vehicle control, DC: diabetic control with streptozotocin administration only, CR control: carbohydrate restricted diet treatment in normal rats, CR + STZ: carbohydrate restricted diet treatment in streptozotocin administered rats. Data are shown as mean ± SD. A P-value less than 0.05 (*P* < 0.05) was considered statistically significant. * Vs Normal control and vehicle control, ^@^ Vs Diabetic control, ^#^ Vs CR control

Streptozotocin treated diabetic control group showed a significant increase of kidney weight/body weight ratio compared to all other study groups. Both carbohydrate restriction control and carbohydrate restricted diabetic rat groups exhibited significantly higher kidney weight/body weight ratio (23%, *P* < 0.01; 63%, *P* < 0.001) as seen against normal control group ratio (Fig. 3). Carbohydrate restriction in streptozotocin treated group significantly lowered kidney weight/body weight ratio by 27% (*p* < 0.001) compared to diabetic control group alone. Moreover a significant difference (*P* < 0.001) of kidney/body weight ratio between carbohydrate restricted control and carbohydrate restricted streptozotocin treated groups was also observed in the present study (Fig. 3).

### Effects of carbohydrate restriction on blood glucose levels in diabetic rats

**Fig. 4:**
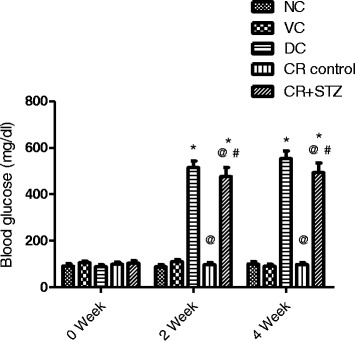
Effects of carbohydrate restricted diet on blood glucose levels in streptozotocin induced diabetic rats at 0, 2, 4 weeks of treatment. NC: normal control, VC: vehicle control, DC: diabetic control with streptozotocin administration only, CR control: carbohydrate restricted diet treatment in normal rats CR + STZ: carbohydrate restricted diet treatment in streptozotocin administered rats. Data are shown as mean ± SD. A *p*-value less than 0.05 (*P* < 0.05) was considered statistically significant. * Vs Normal control and vehicle control, ^@^ Vs Diabetic control, ^#^ Vs CR control

At the baseline no significant changes were observed in blood glucose levels among various study groups. On second week**,** diabetic and carbohydrate restricted diabetic rat groups showed a markedly significant rise in blood glucose levels compared to normal control, vehicle control and carbohydrate restricted control groups (Fig. 4). Besides presence of high blood glucose levels in both diabetic control and carbohydrate restricted diabetic rat groups, the later showed a significantly lower blood glucose level by 7.39% (*P* < 0.05) compared to diabetic control group at second week of study period (Fig. 4). This difference in hyperglycemia levels between carbohydrate restricted control and carbohydrate restricted diabetic rat groups was further noted to be significantly increased to 10.68% (*P* < 0.001) at fourth week of study period.

### Effects of carbohydrate restriction on renal function parameters in diabetic rats

In the present study**,** streptozotocin treated diabetic control group developed a high serum blood urea nitrogen levels compared with all other study groups. Carbohydrate restriction exposure in streptozotocin treated group significantly lowered BUN levels by 41% (*P* < 0.001) from diabetic control group levels (Table 2). Moreover diabetic control group also resulted in significant elevation of serum creatinine levels compared to all other study groups. Carbohydrate restriction intervention in diabetic group restored the serum creatinine levels to normalcy. The serum albumin levels was found to be reduced among diabetic control 44% (*P* < 0.001), carbohydrate restriction control 35% (*P* < 0.001) and carbohydrate restricted streptozotocin treated 57% (*P* < 0.001) rat groups compared to normal control group (Table 2). Streptozotocin administration in diabetic control group induced severe albuminuria and proteinuria compared to all other study groups. Carbohydrate restriction exposure in diabetic rat group reduced urinary albumin and protein excretion to normal range (Table 2).

**Table 2:** Results are expressed as mean ± SD and analysed using one way ANOVA followed by bonferroni multiple comparison test for blood urea nitrogen, serum creatinine, serum albumin, albuminuria and proteinuria levels

	Normal Control	Vehicle Control	Diabetic Control	CR Control	CR + STZ
Serum Parameters					
BUN (mg/dl)	21 ± 2.24	17.6 ± 2.9	64.3 ± 8.4^a^	34 ± 7.2^a,b^	37.8 ± 6.6^a,b^
Creatinine (mg/dl)	0.89 ± 0.141	0.9750 ± 0.119	1.73 ± 0.295^a^	0.94 ± 0.064^b^	1.17 ± 0.106^b^
Albumin (mg/dl)	4.47 ± 0.418	4.1 ± 0.287	2.46 ± 0.691^a^	2.9 ± 0.519^a^	1.91 ± 0.218^a#^
Urine parameters					
Albumin (mg/24 h)	77.00 ± 8.40	71 ± 9.70	551 ± 69^a^	68 ± 11.10^b^	78 ± 10^b^
Protein (mg/24 h)	212 ± 60.10	223 ± 49.90	1351 ± 138.10^a^	116.0 ± 41.40^b^	115.20 ± 38.70^b^

Carbohydrate restriction control (CR control), carbohydrate restriction + streptozotocin treated (CR + STZ). ^a^Vs Normal and vehicle control, ^b^Vs Diabetic control, ^#^ Vs CR control. *P*-value less than 0.05 (*P* < 0.05) was considered statistically significant.

### Effects of carbohydrate restriction on TBARS, antioxidant enzymes and HIF-1α levels in renal tissue of diabetic rats

**Fig. 5:**
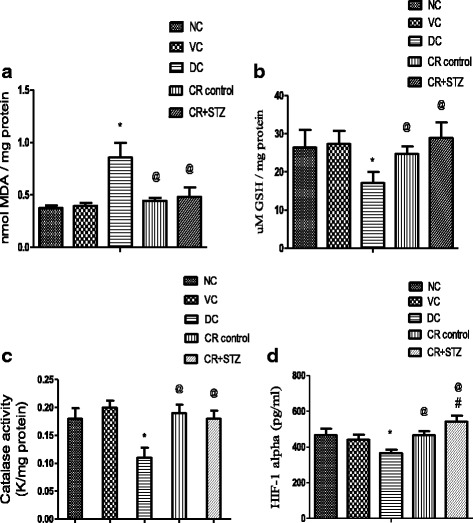
Effects of carbohydrate restricted diet on MDA (malonaldehyde) levels (**a**), reduced glutathione levels (**b**), Catalase activity (**c**) and HIF-1α levels (**d**) in kidney homogenates of streptozotocin induced diabetic rats after 4 weeks. Results are expressed as mean ± SD and analysed using one way ANOVA followed by bonferroni multiple comparison test * *P* < 0.05 Vs Normal and vehicle control, ^@^ *P* < 0.05 Vs diabetic control, ^#^ *P* < 0.05 Vs CR control

Streptozotocin treatment resulted in a significant increase in renal tissue malonaldehyde levels in diabetic control group (56%, *P* < 0.001) compared to normal control rat group levels (Fig. 5a). Carbohydrate restricted diet intake in diabetic rats significantly reduced renal tissue malonaldehyde levels equivalent to normal control group levels. Diabetic control group showed significantly lower 34% (*P* < 0.01) renal tissue reduced glutathione levels compared to normal control group (Fig. 5b). Carbohydrate restriction control and carbohydrate restricted streptozotocin treated rat group exhibited significantly improved renal tissue reduced glutathione levels by 44.15% (*P* < 0.05) and 68% (*P* < 0.001) as seen against diabetic control group. Catalase enzyme activity was observed to be significantly lower in diabetic control group compared to all other study groups. Carbohydrate restriction control (CR control) and carbohydrate restricted diabetic rat groups showed catalase activity equivalent to normal control group (Fig. 5c). Moreover the diabetic kidneys displayed significantly lower hypoxia inducible factor-1 alpha (HIF-1alpha) levels in comparison to various other study groups (Fig. 5d). Conversely carbohydrate restriction exposure in diabetic rat group significantly elevated renal HIF-1α levels by 47.81% (*P* < 0.001), 16.01% (*P* < 0.01) as observed against diabetic control and normal control groups.

### Effect of carbohydrate restriction on histological findings in diabetic rat kidney

**Fig. 6:**
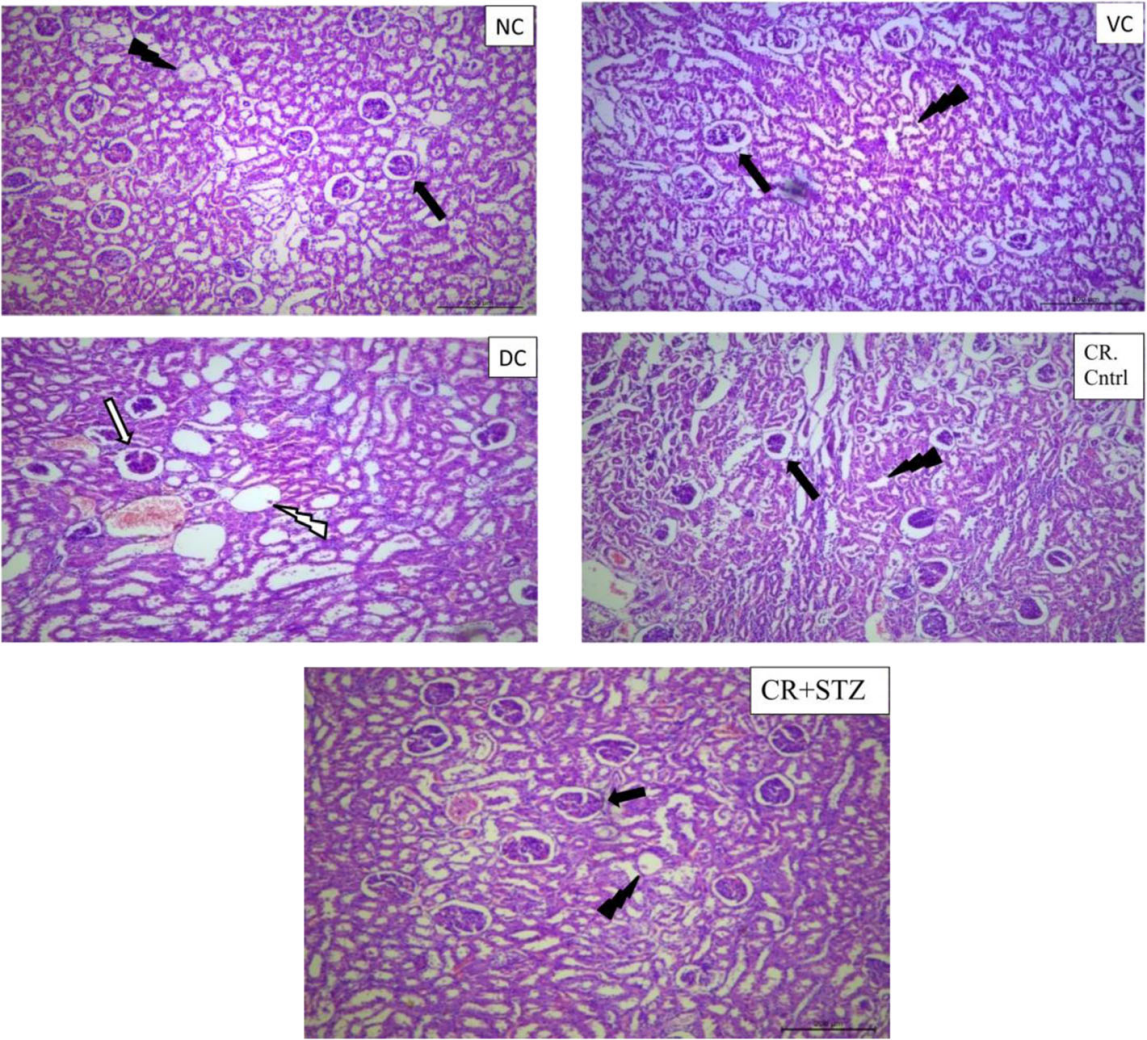
Hematoxylin and eosin staining of kidney sections after 4 weeks of NC: normal control, VC: vehicle control, DC: diabetic control with streptozotocin administration only, CR control: carbohydrate restricted diet treatment in normal rats, CR + STZ: carbohydrate restricted diet treatment in streptozotocin administered rats. NC, VC representing normal glomerular architecture () and tubular space (). The diabetic control (DC) kidney section showing glomerular swelling () marked tubular vacoulation () and degenerative changes. Carbohydrate restricted diet treatment in diabetic rat prevented glomerular enlargement and tubular dilation (CR + STZ), Magnification 100X

**Fig. 7:**
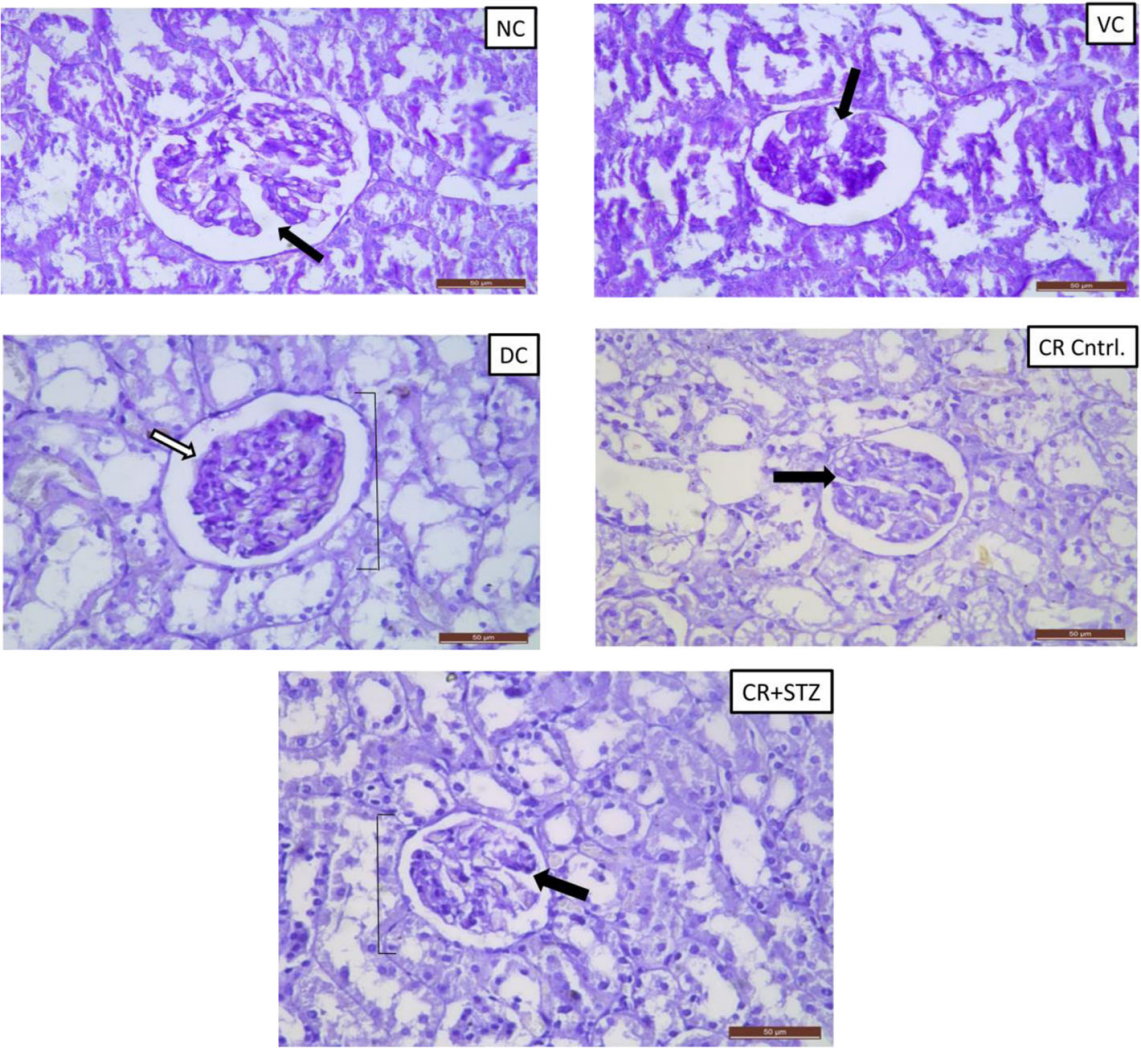
Representive periodic acid schiff (PAS) staining in renal tissues of NC: normal control, VC: vehicle control, DC: diabetic control with streptozotocin administration only, CR control: carbohydrate restricted diet treatment in normal rats CR + STZ: carbohydrate restricted diet treatment in streptozotocin administered rats. The diabetic control rats developed more severe glomerular lesion such as glomerular hypertrophy, global mesangial expansion () white arrow, basement membrane thickening. The absence of glomerular and mesangial alterations in carbohydrate restricted diet treated diabetic kidney section () black arrow. Magnification: 400X

In the present study diabetes induced renal morphological alterations were visualised **as** increased glomerular swelling, tubular dilation and tubular epithelial cell degeneration (Fig. 6, diabetic control group DC). Carbohydrate restriction exposure completely prevented these histological alterations induced by streptozotocin administration in rats (Fig. 6, CR + STZ group). Kidney sections of normal control, vehicle control, carbohydrate restriction control group (NC, VC, CR + STZ) showed normal glomeruli capsular space, no tubular vacoulations and degenerative changes. Periodic acid schiff staining revealed glomerular hypertrophy, proliferation of mesangial cells, and thickening of glomerular basement membrane in diabetic kidney sections (Fig. 7, Diabetic control DC). Carbohydrate restriction exposure completely prevented diabetes induced glomerular injury and structural changes (Fig. 7, CR + STZ group).

## Discussion

Streptozotocin selectively damages insulin-producing pancreatic endocrine cells and can induce experimental hyperglycemia. Sustained hyperglycemia activates complex mechanisms that elevate oxidative stress resulting in renal complications including structural and functional changes [17]. The renal hypoxia has been suggested as link between the oxidative stress and end stage renal disease. Diabetes induced oxidative stress can reduce renal oxygen tension (PO_2_) by impairing tubular electrolyte transport efficiency for Na^+^ reabsorption and mitochondrial uncoupling, these events further lead to the imbalance between metabolic demand and oxygen supply resulting in intrarenal tissue hypoxia [17]. Normally kidneys respond to hypoxic state by up regulating the HIF regulated genes such as erythropoietin, vascular endothelial growth factor or hemeoxygenase-1 [23]. However in diabetic kidneys **HIF** activation may be inadequate resulting in proteinuria, tubulointerstitial fibrosis and end stage renal diseases [4, 5]. Carbohydrate restriction has been documented to exert beneficial effects on various types of kidney disease, and also protect against cancer [24, 25]. In addition a study has reported that low carbohydrate ketogenic diet was able to protect against streptozotocin (STZ) induced pancreatic β cell damage in rats [26]. Kume et al., 2010 [12] showed that long term calorie restriction in mice abolished aging associated renal hypoxia by up regulation of Sirt1 (NAD-dependent histone deacetylase). However the information regarding the effect of carbohydrate restricted diet on type-1 diabetes mediated renal hypoxia which is a major pathogenic event initiating nephropathy but its possible outcomes on renal functional but histological alterations is not known.

Reduction in body weight occurs in type-1 diabetic rats due to excessive protein degradation and loss which was observed after 2 weeks of STZ administration in diabetic control group of present study. Carbohydrate restriction treatment in diabetic group resulted in a markedly significant weight loss despite the absence of proteinuria. Renal hypertrophy is major pathogenic hallmark of diabetic nephropathy and reductions in kidney weight is considered as an important feature of interventions that ameliorate diabetic nephropathy. In our findings kidney weight/body weight ratio was found to be higher in diabetic control rats. Intake of carbohydrate restricted diet in STZ treated rats caused marked reductions in kidney/body weight ratio indicating the beneficial effect of carbohydrate restriction on renal hypertrophy in diabetes. Moreover in the present study carbohydrate restricted diet treatment significantly lowered the elevated blood glucose levels in diabetic rats but these levels remained significantly higher as compared to normal control group. These observations suggest that carbohydrate restricted diet might exert beneficial effects on diabetic kidneys beyond anti-hyperglycaemic action also. Rise in BUN and serum creatinine level occurs in diabetes due to renal insufficiency originating from structural alterations occurring in glomerulus and tubules [27, 28]. Our findings are in line with these previous studies as we also observed similar pattern of elevated serum creatinine and BUN levels in diabetic rats. Carbohydrate diet restriction significantly attenuated the rise in serum creatinine and BUN levels in STZ treated rats. The results of present study also demonstrated low serum albumin levels in diabetic control group which may be a case due to renal insufficiency leading to loss of proteins [29], and/or due to metabolic disturbance resulting in increased protein catabolism [30] or insulin deficiency [31]. Surprisingly**,** low serum albumin levels were also observed in CR control and CR + STZ rat groups without any significant proteinuria. These effects could be explained partially by the shift of metabolic demand for energy towards non carbohydrate substrate for energy like proteins, which in this case may be less available for synthesis of albumin or low insulin levels. Proteinuria develops in diabetic kidney diseases predominantly due to disturbances in the glomerular filtration barrier (podocyte injury) resulting in leakage of proteins in urine [32]. Similarly in the present study diabetic rats displayed massive 24 h proteinuria from second week progressing to fourth week period. Carbohydrate diet restriction treatment completely abolished the raised proteinuria levels in diabetic rats. These effects may be likely due to the reduced damage of the glomerular filtration barrier in type-1 diabetic rats subjected to the carbohydrate restricted diet.

Hyperglycemia induced oxidative stress in diabetes can lead to depletion of tissue antioxidant levels [33] which can result in mitochondrial damage, peroxidation of cell membrane lipids, apoptosis [34]. The vulnerability of renal tissue to glucotoxicity can be prominently mediated by excessive generation of superoxide radicals (O_2_^−^). The enzyme superoxide dismutase (SOD) rapidly catalyses the conversion of O_2_^−^ to hydrogen peroxide (H_2_O_2_) which is further inactivated by catalase [35]. Importantly the catalase deficiency was demonstrated to exacerbate renal injury in diabetic mice [36]. Several studies have shown that calorie restriction can reduce aging associated increase in mitochondrial ROS production and oxidative stress derived damage to proteins and lipids [37–39]. Another study, has demonstrated that carbohydrate restricted diet can increase stress resistance in rats exposed to exhaustive exercise and prevent oxidative damage in brain [10].

In the present study diabetic kidneys displayed lower reduced glutathione (GSH) content, catalase activity and high MDA levels which are indicative of increased oxidative stress. Carbohydrate restricted diet intervention in diabetic rats reduced oxidative stress by elevating the renal tissue GSH levels, catalase activity and thus prevented the formation of tissue lipid peroxides. Moreover diabetic rats exhibited low levels of hypoxia inducible factor (HIF) in renal tissue which might have resulted in renal hypoxia, the final common pathogenic event ensuing the nephropathy. The results from present study demonstrated that carbohydrate restriction by up regulating the renal tissue HIF-1 alpha levels opposed the development of renal hypoxia in diabetic rats. Interestingly, a study has shown that administration of superoxide radical scavenger tempol for 2–14 days enhanced the HIF-1α levels in diabetic kidneys [40]. In addition the acute treatment with tempol was reported to improve the intrarenal tissue hypoxia by countering the diabetes induced superoxide O_2_ˉ generation [9]. Thus it might be possible that in the present study the carbohydrate restricted diet prevented the renal hypoxia by augmenting the antioxidant defense and thereby leading to the HIF-1α activation.

Histological findings from hematoxylin and eosin (H & E) staining revealed marked structural alterations as visualised by glomerular swelling, tubular vacuolation and degenerative changes in diabetic kidney sections. Whereas**,** mesangial expansion and glomerular hypertrophic changes in kidney sections from streptozotocin induced diabetic group were confirmed by periodic acid Schiff (PAS) staining. The results of the present study showed that the intake of carbohydrate energy restricted diet attenuated any glomerular and tubular changes in kidneys of diabetic rats.

Thus carbohydrate restricted diet intake resulted in a marked decline of body weight at fourth week period but improved the kidney/body weight ratio in diabetic rats. Moreover the dietary intervention treatment reduced the levels of oxidative stress indicator TBARS by enhancing the reduced glutathione levels and catalase activity in diabetic kidneys. Diabetes induction resulted in renal hypoxia as observed by the lower levels of HIF-1α found in kidneys of streptozotocin treated rats. Carbohydrate restricted diet treatment upregulated the levels of renal HIF-1α in diabetic rats and prevented the occurrence of hypoxic stress. Further this antioxidant defense activation and HIF-1α upregulation abolished the renal functional and structural alterations in diabetic rat kidneys.

## Conclusion

Our results demonstrated that type-1 diabetic rats fed 30% reduced amount of carbohydrate-energy-restricted (CR) diet exhibited renoprotective effects compared to diabetic rats fed *ad libitium* normal control diet. The restricted diet treatment reduced oxidative stress and prevented occurrence of hypoxia in diabetic kidneys by enhancing tissue antioxidant enzyme and HIF-1 α levels. Furthermore histological findings from kidney sections from CR treated diabetic rats showed absence of glomerular hypertrophy, mesangial proliferation and tubular changes. Thus we conclude that carbohydrate-energy-restricted diet might be a useful therapeutic approach for prevention of nephropathy in type-1 diabetes. Further studies are required to explore the impact of carbohydrate diet restriction on various other molecular signals regulating the HIF-1α levels in diabetic rat kidneys.

## Abbreviations

AIN-93 M carbohydrate: American institute of nutrition-93 maintainence carbohydrate
ANOVA: Analysis of variance
ATP: Adenosine tri phosphate
B.wt: Body weight
CR + STZ: Carbohydrate restriction in streptozotocin treated group
GSH: Reduced glutathione
HIF-1α: Hypoxia inducible factor-1 alpha
HIFs: Hypoxia inducible factors
LPO: Lipid peroxidation
PO_2_: Oxygen tension
QO_2_: Oxygen consumption
ROS: Reactive oxygen species
STZ: Streptozotocin
TBARS: Thiobarbituric acid reactive substances

## References

[1] Larkins RG, Dunlop ME (1992). The link between hyperglycaemia and diabetic nephropathy. Diabetologia.

[2] Welch WJ, Baumgärtl H, Lübbers D, Wilcox CS (2001). Nephron pO2 and renal oxygen usage in the hypertensive rat kidney. Kidney Int.

[3] Friederich M, Hansell P, Palm F (2009). Diabetes, oxidative stress, nitric oxide and mitochondria function. Curr Diabetes Rev.

[4] Manotham K, Tanaka T, Matsumoto M, Ohse T, Inagi R, Miyata T, Kurokawa K, Fujita T, Ingelfinger JR, Nangaku M (2004). Transdifferentiation of cultured tubular cells induced by hypoxia. Kidney Int.

[5] Ohtomo S, Nangaku M, Izuhara Y, Takizawa S, de Strihou CVY, Miyata T (2008). Cobalt ameliorates renal injury in an obese, hypertensive type 2 diabetes rat model. Nephrol Dial Transplant.

[6] Friederich-Persson M, Thörn E, Hansell P, Nangaku M, Levin M, Palm F (2013). Kidney hypoxia, attributable to increased oxygen consumption, induces nephropathy independently of hyperglycemia and oxidative stress novelty and significance. Hypertension.

[7] Semenza GL, Wang GL (1992). A nuclear factor induced by hypoxia via de novo protein synthesis binds to the human erythropoietin gene enhancer at a site required for transcriptional activation. Mol Cell Biol.

[8] Leonard MO, Cottell DC, Godson C, Brady HR, Taylor CT (2003). The role of HIF-1α in transcriptional regulation of the proximal tubular epithelial cell response to hypoxia. J Biol Chem.

[9] Nordquist L, Friederich-Persson M, Fasching A, Liss P, Shoji K, Nangaku M, Hansell P, Palm F (2015). Activation of hypoxia-inducible factors prevents diabetic nephropathy. J Am Soc Nephrol.

[10] De Oliveira SL, Diniz DB, Amaya-Farfan J (2003). Carbohydrate–energy restriction may protect the rat brain against oxidative damage and improve physical performance. Br J Nutr.

[11] Robertson LT, Treviño-Villarreal JH, Mejia P (2015). Protein and calorie restriction contribute additively to protection from renal ischemia reperfusion injury partly via leptin reduction in male mice. J Nutr.

[12] Kume S, Uzu T, Horiike K, Chin-Kanasaki M, Isshiki K, Araki SI, Sugimoto T, Haneda M, Kashiwagi A, Koya D (2010). Calorie restriction enhances cell adaptation to hypoxia through Sirt1-dependent mitochondrial autophagy in mouse aged kidney. The J Clin Invest.

[13] Figgers CL, White TC, Ugochukwu NH (2015). The reno-protective effects of dietary caloric restriction against oxidative damage and inflammation in streptozotocin-induced diabetic rats. NJTR.

[14] Grieb P, Kłapcińska B, Smol E, Pilis T, Pilis W, Sadowska-Krępa E, Sobczak A, Bartoszewicz Z, Nauman J, Stańczak K, Langfort J (2008). Long-term consumption of a carbohydrate-restricted diet does not induce deleterious metabolic effects. Nutr Res.

[15] Feinman RD, Pogozelski WK, Astrup A, Bernstein RK, Fine EJ, Westman EC, Accurso A, Frassetto L, Gower BA, McFarlane SI, Nielsen JV (2015). Dietary carbohydrate restriction as the first approach in diabetes management: critical review and evidence base. Nutrition.

[16] Reeves PG, Nielsen FH, Fahey GC (1993). AIN-93 purified diets for laboratory rodents: final report of the American Institute of Nutrition ad hoc writing committee on the reformulation of the AIN-76A rodent diet. J Nutr.

[17] Palm F, Cederberg J, Hansell P, Liss P, Carlsson PO (2003). Reactive oxygen species cause diabetes-induced decrease in renal oxygen tension. Diabetologia.

[18] Zheng M, Ye S, Zhai Z (2009). Rosiglitazone protects diabetic rats against kidney disease through the suppression of renal moncyte chemoattractant protein-1 expression. J Diabetes Complicat.

[19] Lowry OH, Rosebrough NJ, Farr AL, Randall RJ (1951). Protein measurement with the Folin phenol reagent. J Biol Chem.

[20] Ohkawa H, Ohishi N, Yagi K (1979). Assay for lipid peroxides in animal tissues by thiobarbituric acid reaction. Anal Biochem.

[21] Boyne AF, Ellman GL (1972). A methodology for analysis of tissue sulfhydryl components. Anal Biochem.

[22] Gwinner W, Deters-Evers U, Brandes RP, Kubat B, Koch KM, Pape M, Olbricht CJ (1998). Antioxidant-oxidant balance in the glomerulus and proximal tubule of the rat kidney. J Physiol.

[23] Michiels C (2004). Physiological and pathological responses to hypoxia. Am J Pathol.

[24] Reisin E, Azar S, De Boisblanc BP, Guzman MA, Lohmann T (1993). Low calorie unrestricted protein diet attenuates renal injury in hypertensive rats. Hypertension.

[25] Klement RJ, Kämmerer U (2011). Is there a role for carbohydrate restriction in the treatment and prevention of cancer?. Nutr Metab.

[26] Al-Khalifa A, Mathew TC, Al-Zaid NS, Mathew E, Dashti H (2011). Low carbohydrate ketogenic diet prevents the induction of diabetes using streptozotocin in rats. Exp Toxicol Pathol.

[27] Cohen T, Nahari D, Cerem LW, Neufeld G, Levi BZ (1996). 1996. Interleukin 6 induces the expression of vascular endothelial growth factor. J Biol Chem.

[28] Montilla P, Barcos M, Munoz MC, Bujalance I, Munoz-Castaneda JR, Tunez I (2005). Red wine prevents brain oxidative stress and nephropathy in streptozotocin-induced diabetic rats. J Biochem Mol Biol.

[29] Mauer SM, Steffes MW, Brown DM (1981). The kidney in diabetes. Am J Med.

[30] Almdal TP, Vilstrup H (1988). Strict insulin therapy normalises organ nitrogen contents and the capacity of urea nitrogen synthesis in experimental diabetes in rats. Diabetologia.

[31] Peavy DE, Taylor JM, Jefferson LS (1978). Correlation of albumin production rates and albumin mRNA levels in livers of normal, diabetic, and insulin-treated diabetic rats. P Natl A Sci.

[32] Jefferson JA, Shankland SJ, Pichler RH (2008). Proteinuria in diabetic kidney disease: a mechanistic viewpoint. Kidney Int.

[33] Blum J, Fridovich I (1985). Inactivation of glutathione peroxidase by superoxide radical. Arch Biochem Biophys.

[34] Wang J, Jin W, Zhang W, Hou Y, Zhang H, Zhang Q (2013). Hypoglycemic property of acidic polysaccharide extracted from Saccharina japonica and its potential mechanism. Carbohyd Polym.

[35] Chance B, Sies H, Boveris A (1979). Hydroperoxide metabolism in mammalian organs. Physiol Rev.

[36] Hwang I, Lee J, Huh JY, Park J, Lee HB, Ho YS, Ha H (2012). Catalase deficiency accelerates diabetic renal injury through peroxisomal dysfunction. Diabetes.

[37] Jeon TI, Lim BO, Yu BP, Lim Y, Jeon EJ, Park DK (2001). Effect of dietary restriction on age-related increase of liver susceptibility to peroxidation in rats. Lipids.

[38] López-Torres M, Gredilla R, Sanz A, Barja G (2002). Influence of aging and long-term caloric restriction on oxygen radical generation and oxidative DNA damage in rat liver mitochondria. Free Radical Bio Med.

[39] Pamplona R, Portero-otín M, Requena J, Gredilla R, Barja G (2002). Oxidative, glycoxidative and lipoxidative damage to rat heart mitochondrial proteins is lower after 4 months of caloric restriction than in age-matched controls. Mech Ageing Dev.

[40] Rosenberger C, Khamaisi M, Abassi Z, Shilo V, Weksler-Zangen S, Goldfarb M, Shina A, Zibertrest F, Eckardt KU, Rosen S, Heyman SN (2008). Adaptation to hypoxia in the diabetic rat kidney. Kidney Int.

